# Ethics of iPSC-Based Clinical Research for Age-Related Macular Degeneration: Patient-Centered Risk-Benefit Analysis

**DOI:** 10.1007/s12015-014-9536-x

**Published:** 2014-06-29

**Authors:** Mariko Nakano-Okuno, B. Rashmi Borah, Ichiro Nakano

**Affiliations:** 1 Department of Internal Medicine, The Ohio State University College of Medicine, B054 Graves Hall, 333 W 10th Ave, Columbus, OH 43210 USA; 2 Center for Bioethics and Medical Humanities, Wexner Medical Center, The Ohio State University, Columbus, OH 43210 USA; 3 Department of Neurological Surgery, Wexner Medical Center, The Ohio State University, Columbus, OH 43210 USA; 4 James Comprehensive Cancer Center, Wexner Medical Center, The Ohio State University, Columbus, OH 43210 USA

**Keywords:** Human subject research, Induced pluripotent stem cells (iPSCs), Age-related macular degeneration (AMD), Patient-centered risk-benefit analysis, Tumorigenicity, Therapeutic misconception/misestimation

## Abstract

The opportunity to undergo an induced pluripotent stem cell-based autologous transplant can strike patients as a chance for a cure from a debilitating condition with few options for respite. However, when clinical studies of this caliber present themselves, patients and researchers, each with their own set of motives, may find it difficult to take a balanced approach to evaluating them. We present a patient-centered risk-benefit analysis of the iPSC-based clinical research currently underway in Japan, including a survey of in vitro and in vivo tests that support this project, an in-depth discussion of risks, and further elucidation of considerations patients may wish to consider. The arguments presented will assist patients in undertaking a more informed decision-making process.

## Introduction

**Fig. 1:**
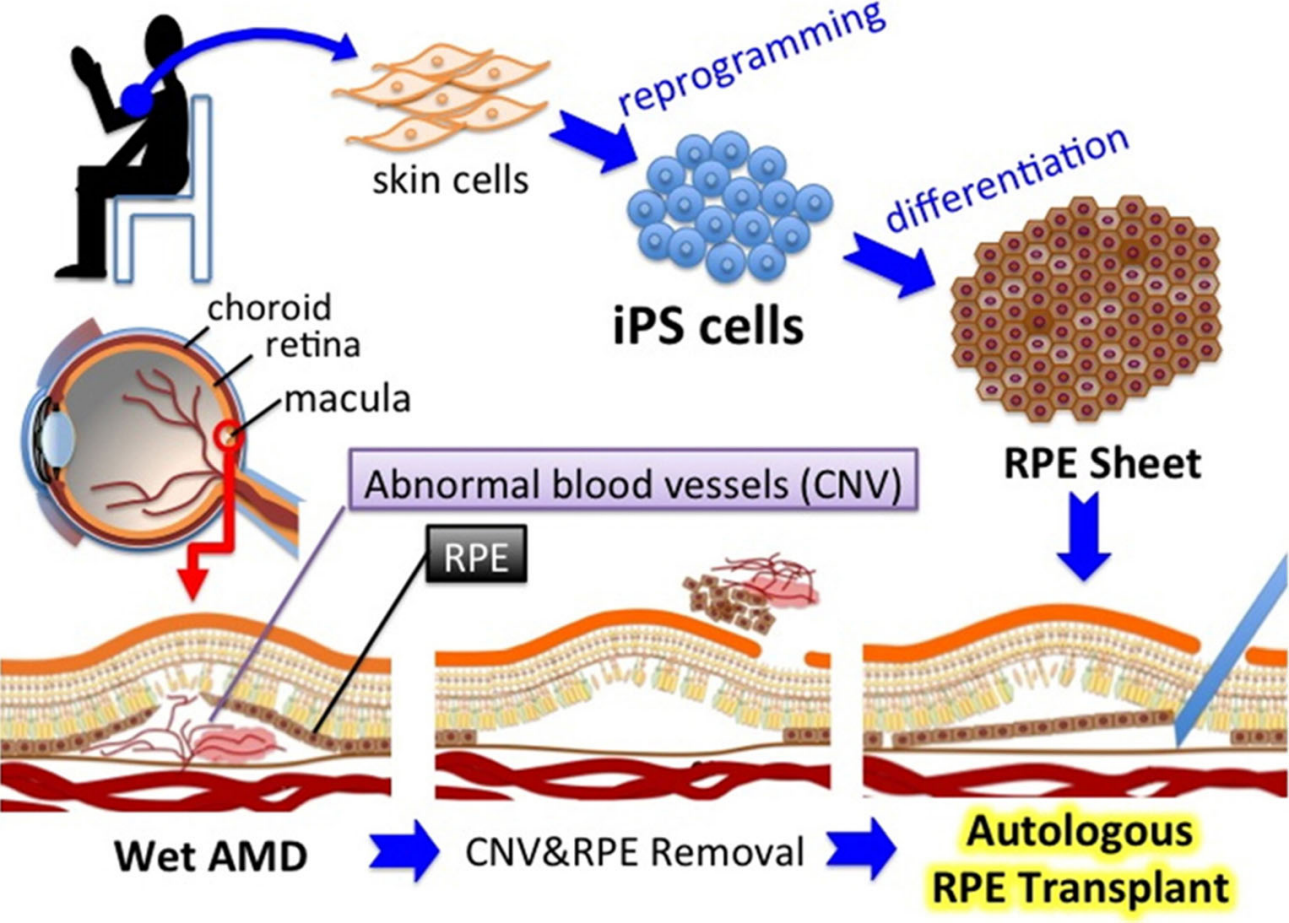
Scheme describing the flow of how iPSCs are generated from a patient’s skin cells and differentiated into RPE cells to be transplanted into the eye of the AMD patient

The launch of the world’s first pilot clinical research to use patient-derived iPSCs for retinal regeneration was officially announced in Japan on July 30, 2013 [1]. This study, which is currently recruiting six patients over the next two to 3 years, targets age-related macular degeneration (AMD) in the exudative form, commonly called wet AMD [2]. Wet AMD is a degenerative retinal disease in which abnormal blood vessels (choroidal neovascularization, CNV) develop in the macula, the region in the back center of the eyeball. This CNV invades retinal pigmental epithelium (RPE) that nourishes and supports photoreceptors, and thereby causes loss of central vision. The idea of the pilot study is to surgically replace the damaged RPE cells with healthy cells developed from patient-derived iPSCs. Researchers will take skin tissue samples (4 mm diameter) from a consenting patient’s upper arm, induce those skin cells into iPSCs by exogenously expressing key pluripotency genes, create sheets of RPE from iPSCs (with the processing time of approx.10 months), cut the RPE sheets into appropriate size (dose not specified) and transplant them under retina after removing CNV under general anesthesia (Fig. 1). The researchers will then investigate possible immune rejection or tumor formation caused by the transplant and other adverse effects and problems during the one-year postoperative period (every month for the first half a year and every 2 months thereafter), with additional 3 years of follow-up. The prospective subjects must be age 50 or over, with refractory or recurrent wet AMD in at least one eye with the corrected visual acuity worse than 20/67 (0.3 in decimals). With this approach, researchers hope to halt retinal degeneration in patients with AMD, though *safety evaluation*, not therapeutic efficacy, of this autologous transplant is the primary focus of the study. Therefore, this study is labeled as a pilot clinical study (*rinsho kenkyu*) instead of a clinical trial that explicitly intends to develop a therapeutic product.

Since Japan’s pilot study is the first iPSC-based autologous transplant on human subjects, its ethical framework will set a precedent for subsequent iPSC-based transplants. Before iPSC-based cell therapy comes into wider clinical use, risk-benefit analyses, informed consent procedures, and ethical review and monitoring processes need to be carefully examined. Although Japan’s iPSC clinical research is not the first attempt to develop stem cell therapy for retinal diseases, as there have already been human clinical trials to transplant hESC- or fetal cell- derived retinal cells for macular degeneration [3, 4], we emphasize the importance of discussing the ethics of iPSC-based clinical research as a separate issue from ethics of hESC-based clinical trials. When compared with hESCs and other cell-based therapy, the iPSCs created from patients’ skin tissues are believed to be genetically identical to each patient’s cells, which could reduce the risk of immune rejection of the transplanted graft by recipient. This may suggest that iPSC-based cell therapy could be a feasible alternative to former transplant attempts. Furthermore, the use of iPSCs is commonly regarded as “more ethical” than the use of hESCs or other embryo-derived cells because the establishment of iPSCs does not involve the use of early human embryos. These two promising features of iPSCs, together with the fact that Dr. Shinya Yamanaka from Japan received the Nobel Prize in 2012 for the successful establishment of iPSCs, excite the Japanese community enough to welcome virtually any ideas of iPSC-based clinical studies. However, it is well documented that there are additional safety and ethical concerns peculiar to clinical applications of iPSCs, as will be discussed later. Researchers must be able to clearly explain such potential concerns to research subjects who are also patients. The ethical challenges of the Japan’s first-in-human iPSC clinical study have been concisely discussed by Habets et al. [5]. Instead of discussing the social value of the clinical study as these authors did, the present paper will focus on the direct risks and benefits to participating patients in order to examine whether these patients could be exploited for the sake of the research’s social value. In addition, Habets et al. do not describe the efforts that the Japanese investigators have made to reduce the study-associated risks. We will appreciate such efforts yet claim that there remains a gap between patients’ and researchers’ perceptions of favorable risk-benefit ratios. The ultimate goal of this paper is not to hail or to denounce this type of research, but to enable the researchers to help their patients make informed choices.

## Patient-Centered Risk Benefit Analysis

**Fig. 2:**
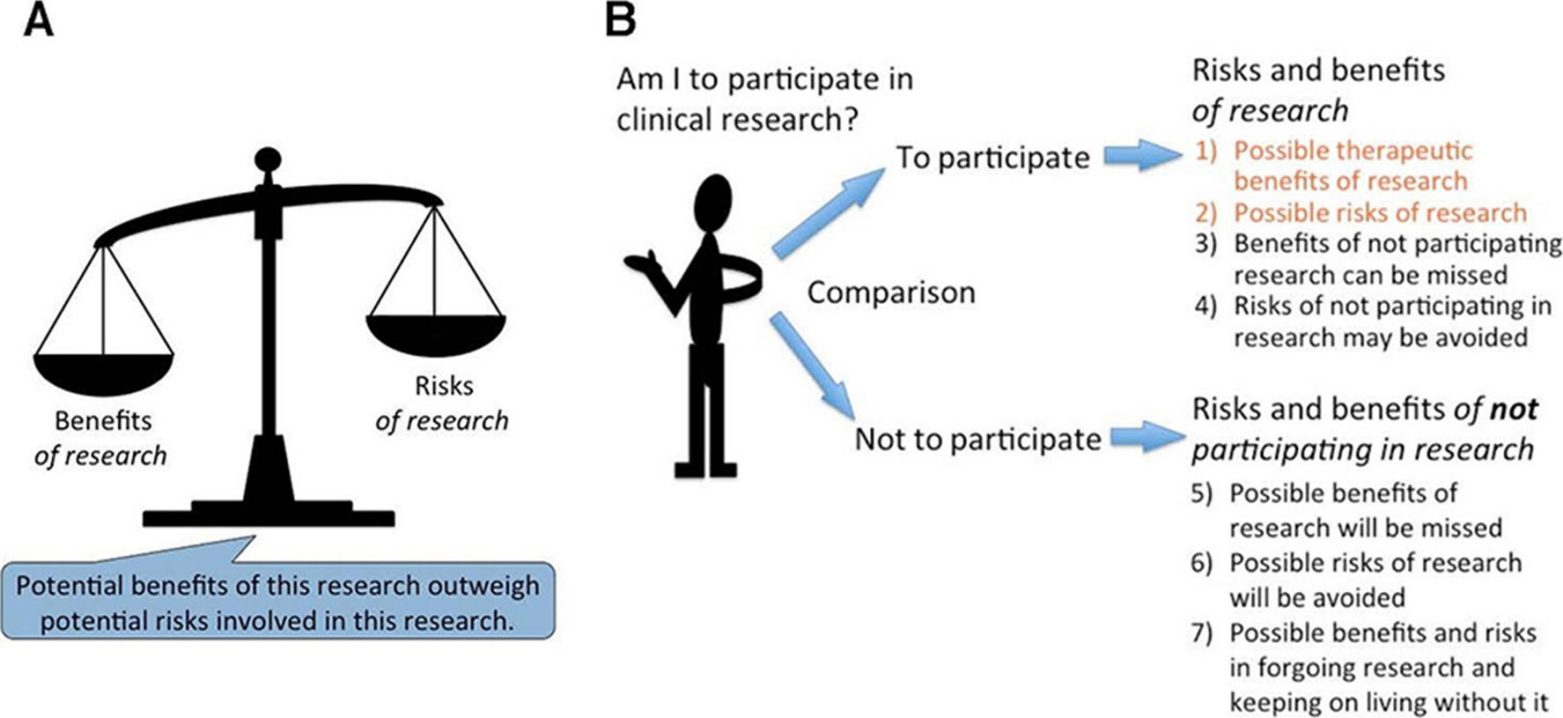
Two Sets of Comparisons. (A) A limited comparison of risks and benefits, as compared to (B) patient-centered decision-making process

An important concept to remember when we conduct such a patient-centered analysis is that the patients’ standpoint is fundamentally different from the researchers’ standpoint. Subsequently, the term “risk-benefit analysis” has a dual meaning depending on which perspective is taken. Though there have been intensive discussions in the field of bioethics on whether we should keep drawing a sharp line between clinical research and clinical practice [6], the Belmont Report’s clear statement that research is an activity quite distinct from clinical practice [7] is still significant and relevant for the purpose of protecting human subjects. The goal of clinical practice is to benefit particular patients by providing the best available treatment and care, whereas the goal of clinical research is to produce generalizable knowledge that may hopefully contribute to future medicine. Given this difference in perceived goals, it is natural for a patient to expect from what he perceives as “an experimental treatment” certain therapeutic benefits, even if that “treatment” is actually *research* conducted in a clinical setting – this phenomenon is widely known as therapeutic misconception [8]. Additionally, the notion of a favorable risk-benefit ratio, as perceived by researchers, might be different from that of patients, which leads to another phenomenon called therapeutic misestimation, or a patient’s tendency to underestimate risks and overestimate benefits of the research. Researchers may think that minor risks to a few patients would be outweighed by possible greater therapeutic benefits to numerous future patients. By contrast, a patient may think that minor risks to one’s health would be outweighed only by possible greater benefits to oneself, not others. Furthermore, when a patient is considering whether to participate in a clinical trial or research, what he or she considers would not only be the comparison between the benefits and harms resulting from participating in research (as shown in Fig. 2a) but also the comparison between alternative options, each of which involves its own set of advantages and disadvantages (Fig. 2b). We should keep in mind that Fig. 2a merely covers the comparison between 1) and 2) in Fig. 2b. To conclude that a certain research is expected to bring about a positive surplus of benefits over risks is *not* to conclude that this surplus outweighs another surplus of benefits over risks of *not* participating in that research. Even if the proposed research’s therapeutic benefits to millions of other patients appear to outweigh relatively small research risks to you, you will have no reason, except for altruistic reasons, to prefer research over non-participation when non-participation is expected to bring you greater overall surplus of benefits over risks than the overall surplus of benefits over risks that the research is expected to bring to you.

This discrepancy between the two parties’ perspectives becomes visible when we take a careful look at how researchers explain their research project to potential participants during the informed consent process. The informed consent document prepared by the Japan’s iPSC-based AMD research group [9] provides a detailed explanation about their research project, covering most items that every human subject research is supposed to cover. It explains who qualifies as research subject, length and duration of the study, the experimental nature and purpose of the study, caution about expected benefits, risks and inconveniences associated with the study, the freedom to withdraw one’s consent at any time without jeopardy, as well as other procedural directions about how to participate in the study. These are items that are commonly mandated by standard informed consent guidelines, such as the Declaration of Helsinki [10] and the Japan Ministry of Health, Labor and Welfare’s Ethical Guideline for Clinical Studies [11]. The same informed consent document also mentions, at least to a moderate extent, the novelty and unpredictability of early iPSC-based clinical study, the tumorigenicity risks of iPSCs as well as the researchers’ plan to compensate research-related health damages, in line with the recommendations set forth in the International Society for Stem Cell Research (ISSCR)’s Guidelines for the Clinical Translation of Stem Cells [12, 13]. However, the researchers’ description of the study does not fully explain several factors that could sway patients’ decisions, and as it stands, it could possibly encourage patients and their families to maintain their therapeutic misestimation. First, the informed consent document would give readers the impression that not all, but most safety concerns are now cleared and that the research is expected to give mild yet positive enough benefits to call the experimental procedure a therapy. It could alarm patients by listing over twenty kinds of possible adverse events that may possibly result from skin tissue sampling, general anesthesia, retinal surgery and iPSC-derived RPE transplant. However, many of such risks seem to be always described in any consent documents for clinical trials. Among the long list of adverse events, one may notice the description about the possibility of tumor formation, or tumorigenicity, of the transplanted cells. Here the informed consent document points out that it is impossible to rule out the risk of tumor formation triggered by the transplanted RPE sheet, since iPSCs, which have the capacity to proliferate indefinitely, are known to form tumors if they are directly transplanted into the body of a living organism. However, the same document continues to argue that there was not even a single case of tumor formation in their preclinical animal studies of iPSC-derived RPE transplant. In another place of the document, it is also explained that the original method to create iPSCs using retroviruses had a high chance of causing abnormalities including tumors, but that such a method is now replaced by a new method using what is called a plasmid, which significantly reduced the tumorigenicity risks of iPSCs. Such explanations will give patients the impression that the investigators’ current method is safe enough. Patients may further be relieved to read the researchers’ assurance that the doctors will carefully monitor all risks and, should any adverse event occur, provide the standard therapy for it. As for the potential benefits of the research, the informed consent document describes that “this study . . . is not expected to bring about remarkable therapeutic effects such as dramatic improvements in visual acuity,” but adds that “the subretinal new blood vessels and exudates will disappear after therapy” and that “it is expected that the retina adjacent to the transplanted new RPE cells will rejuvenate, which would result in the improved brightness in the central vision, prevention of further visual deterioration and, in some cases, slight improvement in visual acuity.” Without understanding how probable those benefits are, and which evidence supports such expectations, patients and their families may have the impression that this research involves mild yet positive effects. Patients could reason, quite legitimately, that this research is most likely to be effective – for, if little therapeutic benefits are expected, what is the point of conducting this study? The autologous feature of this transplant study may make patients optimistic as well – they may well believe that, if their own cells will be used and “put back into them,” it cannot be entirely dangerous.

Second, the RIKEN’s informed consent document explains the risks and benefits of the iPSC-based RPE transplant study in comparison with existing standard therapies, suggesting that none of them are truly effective so far. Given such information alone, patients and their families could believe that iPSC-based RPE transplant is the *only* promising therapy currently available. However, patients need to examine further the information about all options available to them, not limited to current standard therapies, but also information about other emerging non-standard therapies and ongoing clinical trials, and more detailed information of non-treatment options. Prospective subjects, who are also patients, need to consider what the life with the disease will look like, and whether the life with current standard therapy or the life without any treatment will be so miserable that they desperately need to try any new therapy whenever possible.

Third, researchers may tend to assume that a patient is fully informed and is content with the information given to them when the patient asks no questions. In reality, patients may ask few or no questions because they made a bet – simply because they hold onto their therapeutic misestimation based on the limited amount of information given to them.

This is not a researchers’ fault, as the informed consent procedure conforms to major ethical guidelines, and the endeavor to develop safe and effective treatments is a highly regarded mission of clinical scientists. In the following sections, we will make a supplemental endeavor to help researchers (and referring physicians) provide patients with further information on the safety, efficacy and necessity of the proposed pilot study in an attempt to help avoid patients’ therapeutic misestimation and to alleviate miscommunication between researchers and patients.

## Is Science Behind Risks Understood?

Any clinical research involves certain risks. For patients, risks associated with participation in clinical research are worth taking when (1) they can expect that those risks are minor and ignorable and they are convinced that they can contribute to the advancement of medicine with this minor sacrifice of themselves; *or* when (2) they can expect greater therapeutic benefits that compensate research-associated risks and this surplus of benefits over risks is greater than that of other courses of actions they could take.

One feature of the approved iPSC-RPE transplant pilot study is that it is uncertain whether the research-associated risks are truly minor and ignorable. In fact, this is precisely the reason why the primary focus of Japan’s pilot study is the safety of the transplant.

Researchers’ main safety concern is tumorigenicity, or the risk of tumor development, of the iPSCs and the iPSC-derived transplanted grafts [14]. First, “pluripotency,” the potential of iPSCs to differentiate into any type of cells that consist our body, is the hallmark of tumor initiating cells. Similar to ES cells, iPSCs are capable of forming tumors when those cells are injected as immature state into immunocompromised mice. Therefore, if the iPSC-derived grafts contained incompletely differentiated cells, those cells would have the tumorigenic potential. Most of such tumors are benign teratomas, but there are reported cases of malignant germ-cell tumors, such as embryonal carcinoma, being formed [15, 16]. Second, even differentiated pluripotent stem cells could reprogram their cell fate, resulting in de-differentiation into tumorigenic potential, for they may retain or reactivate once-activated yet artificially silenced pluripotency-gene expression pathways inherent to them [14, 17]. Cell-culture-induced differentiation occurs through multiple mechanisms, many of which are epigenetic events, and a majority, if not all, of epigenetic events are reversible. It is true that during the course of differentiation, these cells supposedly lose the capacity to re-acquire the immature stem cell state, but we have to be careful as to whether the cells for clinical use, which should have “differentiated” via in vitro cell culture, indeed commit to irreversible differentiation. Due to the limited studies on this potential dedifferentiation mechanism, the chance of reprogramming event of the differentiated iPS cells into tumorigenic potential remains unknown. Given that the native somatic cells have some risks to become cancers, the open question is whether the cancer-initiating risk of injected “de-differentiated” iPSCs is reasonably lower than the cancer-initiating risks of the native somatic cells.

Another concern with the use of iPSCs is their genomic instability, due to its artificial cell reprogramming process, which may lead to even higher risks of generating tumors than ESCs [18]. In particular, the classic method to produce iPSCs involves the insertion of pluripotency genes (including oncogenic c-Myc) via viral vectors. The use of retroviral vectors allows the integration of pluripotency-initiating genes into the host genome, which may cause mutations in the host cells, or the inserted genes may reactivate the pluripotency network even after the host cell undergoes differentiation. In addition, previous studies have demonstrated the deletion of a number of tumor-suppressor genes in established iPSCs (while those genes were present in the somatic cells of origin), which directly indicates chromosomal instability with *de novo* mutations for tumorigenesis in iPSCs [19].

Researchers have made several attempts to prevent these tumorigenicity-associated risks. They circumvent genome-integrative reprogramming methods by causing only *transient* expression of pluripotency factors to initialize somatic cells, the effect of which gradually gets lost once the iPSCs are established and start replicating themselves [20]. Such non-integrative reprogramming methods include the use of episomal vectors (containing round-shaped DNAs called plasmids that replicate themselves) [21], Sendai virus (a minus strand RNA virus that can infect the somatic cells for a relatively long time) [22], and the use of a synthetic self-replicative RNAs [23] or small molecule compounds alone [24]. Researchers also substitute oncogenic pluripotency genes with different genes that have not been previously known to be involved in tumorigenesis [21, 25]. More recently reported was a simpler method of reprogramming somatic cells into iPSC-like cells by exposing the cells to sublethal external stimuli such as soaking them in the acidic medium, a method that entirely forgoes the introduction of transcription factors [26] —though the veracity of this paper is called into question at this moment, and even if it turned out to be a real phenomenon, it is yet to be ascertained whether this stress-triggering method can be applicable to older adults who suffer from diseases and whether the reprogrammed cells do not gain tumorigenic potential.

With these new advances at hand, the Japanese investigators conducting the iPSC-derived RPE transplant study have adopted one of such non-integrative reprogramming methods, using episomal Epstein-Barr virus (EBV) vectors that carry plasmids encoding transcription factors *without* one of oncogenic transgenes, c-Myc [27, 28]. They have also elaborated the procedure to purify iPSC-derived RPE cells by carefully selecting developed hexagon-shaped brown pigmental cells. They also created RPE *sheets* by putting isolated RPE cells together, so that those cells remain as stable and less likely to transform as possible. In addition, these researchers diligently conducted first through fourth animal tumorigenicity tests over the past 2 years, using over a hundred mice and several macaque monkeys and reported that none of them actually developed tumors after iPSC-derived RPE transplant [27]. (For measures taken by the investigators to reduce research-associated risks, see Table 1.)

**Table 1:** Concerns and countermeasures

	(A) Types of Concern	(B) Measures Taken		(C) Counterarguments
Tumorigenicity	Use of viral vectors that integrate inserted genes into the host genome, thereby causing mutations and encouraging tumorigenic gene expressions	To use non-integrative cell reprogramming method using episomal vectors containing Epstein-Barr-virus (EBV) -based plasmids, which remain in the host cell for 10–14 days for the cell-reprogramming purpose but gradually disappear after multiple cell divisions	Tumorigenicity tests in animals	■ Undeniable chance of integration of a small percentage of plasmids into host genome ■ Oncogenic potential of wile-type woodchuck hepatitis post-transcriptional regulatory element (WPRE), a fragment encoded in the plasmid to facilitate strong expression of reprogramming genes ■ Possible cell proliferation encouraged by the EBV nuclear antigen 1 (EBNA1) gene, which is indispensable for temporal bindings of plasmids to the host chromosomes ■ Tumorigenicity risks unrelated to genomic insertion being unresolved
	Use of oncogenic transcription genes	To substitute c-Myc with alternative factors		
	Malignant transformation of residual undifferentiated cells	To purify RPE cells to eliminate undifferentiated low-grade cells; to conduct product inspection		
	Reactivation of tumorigenic network in differentiated RPE cells	To create an RPE sheet so that the differentiated cells remain stable and hard to transform		
	Immunosuppression encouraging tumor formation	Autologous transplant that may reduce the risk of immune rejection		■ Fundamental question about inherent tumorigenic potential of iPSCs
				■ Possibility of contamination not being ruled out ■ Possibility of de-differentiation of injected cells into tumorigenic immature cells due to microenvironment in vivo
				■ Limited data of macaque monkeys at presentLikelihood and degree of immune rejection yet to be investigated
Graft survival	Immune rejection	Same as above		Same as above
	Low-efficiency in survival of graft cells			■ The more differentiated, the harder the graft cells tend to survive in vivo
Others	Risks associated with eye surgery (retinal detachment, etc.)			
	Viral infection caused by contaminated graft cells	Manufacture in compliance of Good Manufacturing Practice		
	Others			

However, we need to recognize that, regardless of which method to establish iPSCs is used, those artificially reprogrammed pluripotent cells are cells that have risks for tumorigenicity. Even if c-Myc and other oncogenic genes were all replaced by other transcription factors, successful creation of pluripotent stem cells should, by definition, accompany secondary activations of proto-oncogenes including c-Myc. Moreover, when plasmid vectors are used to produce iPSCs, we should note that a small percentage of plasmids are known to actually integrate into the chromosomes of a few host cells [21, 29]. This occasional integration issue may be surmountable, for PCR- based analysis can detect all the integration sites [30], and researchers will at least be able to inspect the established iPSCs and select integration-free clones for clinical applications. Nevertheless, genomic integration is not the only problem related to tumorigenicity. EB virus infection-associated cellular inflammation and subsequent genomic instability can also cause tumors that are unrelated to genomic insertion. To be noted here is that the use of EBV-based episomal plasmid vector requires simultaneous use of supportive genes or elements (woodchuck hepatitis post-transcriptional regulatory element, or WPRE, and the EBV nuclear antigen 1 gene, or EBNA1) that are associated with oncogenic potential [31, 32]. As for such inevitable genomic instability, the Japan’s research panel in the Ministry of Health, Labor and Welfare advised the iPSC-RPE clinical investigators to conduct whole genome sequencing, exome sequencing and CNV analysis on the first several iPSC lines, and the investigators agreed to do so [27]. These analyses will provide us with useful generalizable knowledge about the genomic instability and tumorigenic potential of iPSC-derived cells, but they are not meant to reduce the tumorigenicity risks for the participating patients. There are alternate integration-free reprogramming methods other than the use of EBV-based vectors, such as the RNA- or chemical compound- mediated reprogramming mentioned above, which could eventually turn out to be safer. However, it will take months to test the clinical applicability of those new methods. Whether patients should wait until another reprogramming method is fully developed and clinically applied, or whether it is advisable to go ahead to join the pilot study with the current protocol, would be a meaningful ethical question to discuss.

As for the seemingly favorable preclinical animal tests, we should keep in mind that all the mice used in the study were observed for three to six months before being sacrificed, and the survival of the autologous RPE-transplanted macaque monkey was observed for only 1 year. Thus, long-term tumorigenicity of the transplanted cells in humans has yet to be ascertained. Over time, the microenvironment for injected cells in situ could result in late-onset tumorigenic events. There is a reasonable possibility that the aforementioned non-integrative, non-retroviral cell reprogramming method will only postpone the onset of tumorigenesis, rather than eliminate the tumorigenic potential of the iPSCs.

Granted that the research subjects will be carefully monitored for 1 year with a subsequent three-year follow-up, some patients may understand and accept the risks of tumorigenicity in the hope that this novel treatment strategy will give them sufficient therapeutic benefits. Other patients may think, however, that those are potentially life-threatening risks that are completely avoidable by not participating in the current research project. In response to the seemingly alarmist description of the tumorigenicity risks of iPSCs presented above, researchers may explain to patients that, in worst case scenarios, one can always remove the eye if there are any localized adverse events related to the cell transplant, and that this is exactly one of the reasons why an eye disease was selected for the first-in-human iPSC-based transplant study. However, given the fact that many wet AMD patients continue to maintain peripheral vision of 20/400 and above (as discussed below), some patients would reasonably prefer a 100 % chance of having their minimum vision over a small chance of losing one eye, unless they can expect that the transplant will bring them extra benefits. Our next question is whether these risks can be outweighed by potential therapeutic benefits.

## Potential Benefits?

For patients, any potential benefits of clinical research may be worth pursuing when they can expect that the chance of obtaining such benefits exceeds the benefits from existing treatment or non-treatment options, without suffering from major side effects.

AMD is not a fatal disease, but it affects patient’s quality of life by impacting one’s central vision. According to American Macular Degeneration Foundation, the likely scenario for wet AMD patients would be a life with visual acuity gradually falling down to somewhere between 20/80 and 20/400, but in some cases it could be worse [33].

Current treatment options for wet AMD include (1) monthly anti-VEGF (vascular endothelial growth factor) injections into the eye to block the growth of CNVs and (2) photodynamic therapy or laser surgery to halt the growth of CNVs. However, these are not cures, and the patient’s condition could get worse even with treatment. The National Eye Institute’s Age-Related Eye Disease Study (AREDS) found that (3) the intake of a particular combination of antioxidants and zinc could reduce the risk of worsening the patients’ AMD condition to a certain degree [34]. Despite these attempts, it is true that more effective therapies that can restore vision are anticipated. In February 2013, FDA approved the Argus II Retinal Prosthesis System, an implanted retinal prosthesis (artificial retina) connected to a small video camera and a video processing unit [35], but this approach requires invasive surgery and unwieldy equipment. This is why various cell therapies are being attempted intensively.

Then how much improvement can AMD patients expect with cell therapy? An example closest to the iPSC-RPE transplant would be ESC-derived RPE transplant for macular degeneration in animal models and in a few patients. Putting ethical controversies on the clinical use of ESC-derived cells aside, we can at least predict regenerative medicine’s therapeutic effects on a patient’s vision.

In animal studies, transplantation of primate ESC-derived RPE cells into 4-week-old Royal College of Surgeons (RCS) rats, a widely accepted rodent model of AMD, has reportedly enhanced the survival of the rats’ photoreceptors and improved the visual function [36]. This functional improvement was interpreted by the rats’ frequent head-tracking behavior while watching a rotating black-and-white stripe cylinder 8 weeks after transplantation. Another group reported similar results [37, 38]. This can be seen as a promising sign of effectiveness of the transplant, but these results need to be cautiously interpreted. Using head tracking in rats as readout of evaluation of visual acuity may not be reliable indication of visual function in humans. We should also note that the ages of tested rats do not match the patient population of AMD, for it is obvious that younger mammals can adapt therapeutic benefits more successfully than older mammals. Lastly, in most, if not all, of these studies, scientists use “sham” as control. However, a more appropriate control should be some cells without regenerative capacity, or ideally, cells used in previous clinical trials such as fetal-eye-derived RPE transplant.

**Fig. 3:**
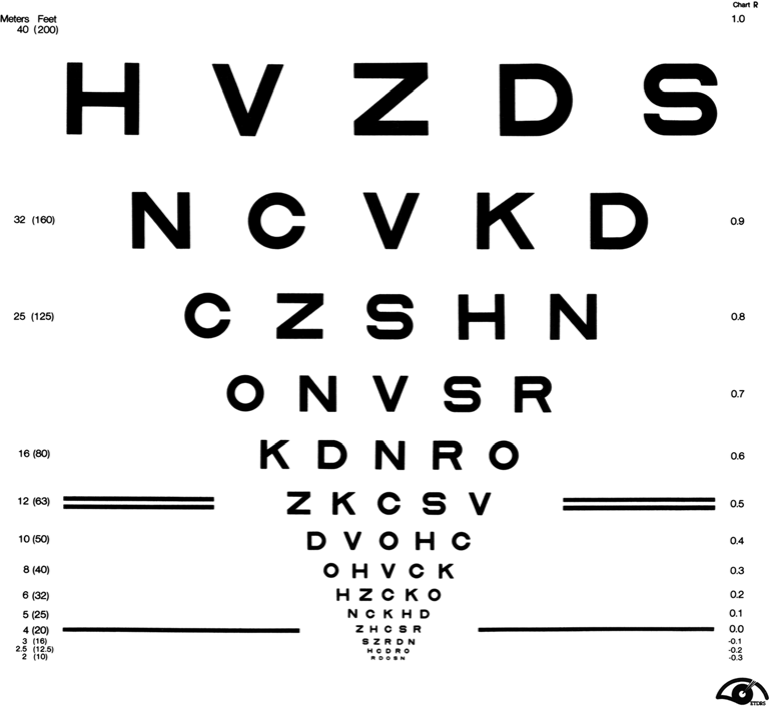
ETDRS Chart (Credit: National Eye Institute, National Institutes of Health) is used to detect subtle changes in a low-vision patient’s visual acuity

In human cases, there are two clinical trials using distinct cells for transplantation, human ESC-derived RPE cells and human-fetal-eye-derived RPE cells, which showed mixed results for AMD patients. The first trial with human ESC-derived RPE cells is still on-going, and has been done with only two patients, one with dry AMD and the other with Stargardt’s, a juvenile macular dystrophy [39]. Outcome for a patient with Stargardt’s has been somewhat encouraging, whereas that for a patient with AMD has been mediocre. On a positive note, although the follow-up was as short as 4 months, both patients showed no sign of tumorigenicity or of immune rejection of the grafts. Following the moderate improvement of the dry AMD patient’s best corrected visual acuity from 21 to 33 letters on the ETDRS chart (Fig. 3, improvement from 20/500 to 20/200) at week 2, it gradually worsened to reading 28 ETDRS letters (20/320) by week 6. Authors claim that the possible reason for this limited improvement is due to the patient’s failure to follow the immunosuppressive regimen for the first week after transplant.

The other trial of human-fetal-eye-derived RPE transplant may endorse the concern that the visual improvement gained can be temporary [40]. In this phase II trial, 7 out of 10 patients (including all 4 dry AMD patients involved) showed improved EDTRS visual acuity scores without showing the sign of graft rejection. Interestingly, there is one case in which a patient (with retinitis pigmentosa, not AMD) showed remarkable visual improvement from seeing nothing but shadows (20/800) to reading large prints and sending emails (20/160) within a year, but even in this case, her visual acuity started to deteriorate after a couple of years and it had dropped down to 20/320 after 6 years. Authors suggest that this temporary visual improvement may not be ascribed to the successful functioning of the transplanted RPE cells, but to the “trophic effect” in which the graft is releasing growth factors to help restore deteriorating host photoreceptors [40, 41]. If this explanation is true, would it not be equally effective and less invasive to directly inject these growth factors into the subretinal spaces of patients’ eyes, rather than to let patients undergo transplant surgery? In any case, sample sizes of these studies were still limited, and further studies are needed to determine the therapeutic efficacy of these replacement therapies.

The inclusion criteria of Japan’s iPSC-RPE clinical research specifies that the qualified patient’s vision should be as wrong as hand motion to 20/67 (0.3 in decimals) and that a patient must have tried the standard wet AMD treatment – at lest four times of anti-VEGF injection treatment – but failed [42]. Given the results of the previous clinical trials, those patients who currently have vision better than the expected from these trials would wish to reexamine the meaning of joining the study. One last point that might be worth considering is that it remains unclear whether and how the iPSC-derived RPE transplant would prevent the neovascularization in the wet AMD patient’s eye and the relapse of the disease. Patients should be aware that the RIKEN’s informed consent document clearly states that such a relapse, or the non-efficacy of the transplant, will not be regarded as “health damage” and hence will not be compensated by the RIKEN and the collaborating hospital.

Getting back to the potential benefits alluded in the informed consent document of the Japan’s clinical study, we propose that the experimental nature of the study and the uncertainty of its therapeutic benefits should be conveyed to participating patients more clearly. When possible therapeutic benefits, such as the improved brightness in the central vision or the improved visual acuity, are mentioned during the informed consent process, researchers and recruiting physicians should give interested patients perspicuous explanations of whether those favorable effects are the direct results of the use of iPSCs or whether the same effects can be caused by any surgery to remove the developed CNVs or by the simple injection of growth factors. Such an articulation would be more ethically appropriate because it will allow patients to carefully compare effects and risks of alternative studies and therapies. Given the excitement and hypes about iPSC research among the Japanese people and the media, a slightly wary explanation of the study may be rather useful for a patient to make a considered decision.

## Conclusion

At times, humans tend to be goal-oriented, and when one realizes the decline in one’s health a fight for recovery is not uncommon. A patient’s desperation can be easily exploited for purposes unrelated to their future well-being. Researchers should be aware of this tendency when alluding to potential benefits.

Some patients may still have reasons to participate in iPSC-based transplant study at this point in time. If one were a patient who aspires to stop the progress of his eye disease now to accomplish tasks that were otherwise impossible on his own, or if one were an elderly patient genuinely wishing to make a small piece of contribution to future medicine at the final stage of his life, one may be ready to undertake the presumably small chances of research-associated risks. Others may simply decide not to participate in research. It is up to individuals. Before they make up their minds, however, research risks and possible benefits have to be examined from various viewpoints and compared with other options. Prior to participation in the study, prospective participants should also be given the opportunity to observe how other AMD patients cope with, or have even overcome, their hardships with various non-therapeutic devices and supports. With or without effective therapies, AMD patients should not live in social isolation without various activities and communications they used to embrace.

## Abbreviations

iPSC: induced pluripotent stem cells
hESC: human embryonic stem cells
AMD: age-related macular degeneration
RPE: retinal pigmental epithelium
CNV: choroidal neovascularization

## References

[1] RIKEN (2013). Press release. http://www.riken.jp/en/pr/press/2013/20130730_1/. Accessed 24 Apr 2014.

[2] RIKEN and Foundation for Biomedical Research and Innovation (2013). Pilot safety study of iPSC-based intervention for wet-type AMD. Available at http://www.riken-ibri.jp/AMD/english/index.html, in Japanese at http://www.riken-ibri.jp/AMD/research/index.html. Accessed 24 Apr 2014.

[3] Ramsden CM, Powner MB, Carr AJ, Smart MJ, da Cruz L, Coffey PJ (2013). Stem cells in retinal regeneration: past, present and future. Development.

[4] Carr AJ, Smart MJ, Ramsden CM, Powner MB, da Cruz L, Coffey PJ (2013). Development of human embryonic stem cell therapies for age-related macular degeneration. Trends in Neuroscience.

[5] Habets MG, van Delden JJ, Bredenoord AL (2014). The inherent ethical challenge of first-in-human pluripotent stem cell trials. Regenerative Medicine.

[6] Solomon, M.Z., & Bonham, A.C. eds. (2013). Ethical oversight of learning health care systems. *Hastings Center Report Special Report,* 43 (1): S1-S44.

[7] U.S. Department of Health, Education, and Welfare (1979). The Belmont Report: ethical principles and guidelines for the protection of human subjects of research.

[8] Horng S, Grady C (2003). Misunderstanding in clinical research: distinguishing therapeutic misconception, therapeutic misestimation and therapeutic optimism. IRB.

[9] RIKEN, Institute of Biomedical Research and Innovation Hospital and Kobe City Medical Center General Hospital (2013). Informed consent document, version 4.8, 28 June 2013. Attached to the final report issued by the Review Panel concerning Human Stem Cell Clinical Research, the Japan Ministry of Health, Labor and Welfare, July 12, 2013. Its simplified version, “Summary for patients,” can also be found at http://www.riken-ibri.jp/AMD/english/summary.html.

[10] World Medical Association (2013). World Medical Association Declaration of Helsinki: ethical principles for medical research involving human subjects, the 2013 version. JAMA.

[11] The Japan Ministry of Health, Labor and Welfare (2008). Ethical guideline for clinical studies. http://www.mhlw.go.jp/general/seido/kousei/i-kenkyu/rinsyo/dl/shishin.pdf.

[12] The International Society for Stem Cell Research (2008). Guidelines for the clinical translation of stem cells. http://www.isscr.org/home/publications/ClinTransGuide. Accessed 24 Apr 2014.

[13] Hyun I, Lindvall O, Ahrlund-Richter L (2008). New ISSCR guidelines underscore major principles for responsible translational stem cell research. Cell Stem Cell.

[14] Lee AS, Tang C, Rao MS, Weissman IL, Wu JC (2013). Tumorigenicity as a clinical hurdle for pluripotent stem cell therapies. Nature Medicine.

[15] The International Stem Cell Initiative (2007). Characterization of human embryonic stem cell lines by the international stem cell initiative. Nature Biotechnology.

[16] Griscelli F, Féraud O, Oudrhiri N (2012). Malignant germ-cell-like tumors, expressing Ki-1 antigen (CD30), are revealed during in vivo differentiation of partially reprogrammed human-induced pluripotent stem cells. American Journal of Pathology.

[17] Dressel R, Schindehütte J, Kuhlmann T (2008). The tumorigenicity of mouse embryonic stem cells and in vitro differentiated neuronal cells is controlled by the recipients’ immune response. PLoS One.

[18] Steinemann D, Göhring G, Schlegelberger B (2013). Genetic instability of modified stem cells – a first step toward malignant transformation?. American Journal of Stem Cells.

[19] Laurent LC, Ulitsky I, Slavin I (2011). Dynamic changes in the copy number of pluripotency and cell proliferation genes in human ESCs and iPSCs during reprogramming and time in culture. Cell Stem Cell.

[20] Yamanaka S (2012). Induced pluripotent stem cells: past, present, and future. Cell Stem Cell.

[21] Okita K, Matsumura Y, Sato Y (2011). A more efficient method to generate integration-free human iPS cells. Nature Methods.

[22] Fusaki N, Ban H, Nishiyama A, Saeki K, Hasegawa M (2009). Efficient induction of transgene-free human pluripotent stem cells using a vector based on Sendai virus, an RNA virus that does not integrate into the host genome. Proceedings of the Japan Academy.

[23] Yoshioka N, Gros E, Li HR (2013). Efficient generation of human iPSCs by a synthetic self-replicative RNA. Cell Stem Cell.

[24] Hou P, Li Y, Zhang X (2013). Pluripotent stem cells induced from mouse somatic cells by small-molecule compounds. Science.

[25] Montserrat N, Nivet E, Sancho-Martinez I (2013). Reprogramming of human fibroblasts to pluripotency with lineage specifiers. Cell Stem Cell.

[26] Obokata H, Wakayama T, Sasai Y (2014). Stimulus-triggered fate conversion of somatic cells into pluripotency. Nature.

[27] Review Panel on Human Stem Cell Clinical Research, the Health Science Council in the Japan Ministry of Health, Labor and Welfare. (2013). On opinions concerning RIKEN’s plan to conduct human stem cell clinical research, 12 Jul 2013. Available in Japanese at http://www.mhlw.go.jp/stf/shingi/2r98520000036w7i-att/2r98520000036x94.pdf. Accessed 24 Apr 2014.

[28] Use of c-Myc was denied by M. Takahashi, Project Leader of the iPSC-RPE transplant study, on Masayo Takahashi Twitter on 8 Mar 2013.

[29] Sommer CA, Montoslavsky G (2010). Experimental approaches for the generation of induced pluripotent stem cells. Stem Cell Research & Therapy.

[30] Okita K, Yamanaka S (2011). Induced pluripotent stem cells: opportunities and challenges. Philosophical Transactions of the Royal Society B: Biological Sciences.

[31] Kingsman SM, Mitrophanous K, Olsen JC (2005). Potential oncogene activity of the Woodchuck hepatitis post-transcriptional regulatory element (WPRE). Gene Therapy.

[32] Frappier L (2012). Contributions of Epstein-Barr nuclear antigen 1 (EBNA1) to cell immortalization and survival. Viruses.

[33] American Macular Degeneration Foundation. http://www.macular.org. Accessed 24 Apr 2014.

[34] U.S. National Eye Institute. http://www.nei.nih.gov/health/maculardegen/armd_facts.asp. Accessed 24 Apr 2014.

[35] U.S. Food and Drug Administration (2013). FDA approves first retinal implant for adults with rare genetic eye disease, 14 Feb 2013. http://www.fda.gov/NewsEvents/Newsroom/PressAnnouncements/ucm339824.htm. Accessed 24 Apr 2014.

[36] Haruta M, Sasai Y, Kawasaki H (2004). In vitro and in vivo characterization of pigment epithelial cells differentiated from primate embryonic stem cells. Investigative Ophthalmology & Visual Science.

[37] Lund RD, Wang S, Klimanskaya I (2006). Human embryonic stem cell-derived cells rescue visual function in dystrophic RCS rats. Cloning Stem Cells.

[38] Jin Z-B, Okamoto S, Mandai M, Takahashi M (2009). Induced pluripotent stem cells for retinal degenerative diseases: a new perspective on the challenges. Journal of Genetics.

[39] Schwartz SD, Hubschman JP, Heilwell G (2012). Embryonic stem cell trials for macular degeneration: a preliminary report. Lancet.

[40] Radtke ND, Aramant RB, Petry HM, Green PT, Pidwell DJ, Seiler MJ (2008). Vision improvement in retinal degeneration patients by implantation of retina together with retinal pigment epithelium. American Journal of Ophthalmology.

[41] Graham-Rowe D (2008). Retinal transplants see fleeting success: but efficacy and practicality of the procedure are called into question. Nature.

[42] RIKEN. (2013). Press release. Available in Japanese at http://www.riken.jp/pr/topics/2013/20130730_1/, Article 1–6. Accessed 24 Apr 2014.

